# Rodent Damage to Natural and Replanted Mountain Forest Regeneration

**DOI:** 10.1100/2012/872536

**Published:** 2012-05-01

**Authors:** Marta Heroldová, Josef Bryja, Eva Jánová, Josef Suchomel, Miloslav Homolka

**Affiliations:** ^1^Institute of Vertebrate Biology, ASCR, Květná 8, 603 65 Brno, Czech Republic; ^2^Institute of Forest Ecology, Faculty of Forestry and Wood Technology, Mendel University in Brno, Zemědělská 3, 613 00 Brno, Czech Republic

## Abstract

Impact of small rodents on mountain forest regeneration was studied in National Nature Reserve in the Beskydy Mountains (Czech Republic). A considerable amount of bark damage was found on young trees (20%) in spring after the peak abundance of field voles (*Microtus agrestis*) in combination with long winter with heavy snowfall. In contrast, little damage to young trees was noted under high densities of bank voles (*Myodes glareolus*) with a lower snow cover the following winter. The bark of deciduous trees was more attractive to voles (22% damaged) than conifers (8%). Young trees growing in open and grassy localities suffered more damage from voles than those under canopy of forest stands (*χ* ^2^ = 44.04, *P* < 0.001). Natural regeneration in Nature Reserve was less damaged compared to planted trees (*χ* ^2^ = 55.89, *P* < 0.001). The main factors influencing the impact of rodent species on tree regeneration were open, grassy habitat conditions, higher abundance of vole species, tree species preferences- and snow-cover condition. Under these conditions, the impact of rodents on forest regeneration can be predicted. Foresters should prefer natural regeneration to the artificial plantings.

## 1. Introduction

Small terrestrial mammals represent an important component in forest ecosystems [1]. In food chains, they function as consumers of primary and secondary production. Thus, they often compete with the interests of forest regeneration and silviculture. According to data from the Ministry of Agriculture (2004), for the period 1994–2004, some 1200 ha of reforested area per year have been damaged by rodents in the Czech Republic.

Densities of small mammals can fluctuate widely. Species with high potential growth rates and populations living in seasonal environments are subject to particularly marked fluctuations in abundance and cause the damage on crops or forest plantations [2–6]. These changes are dictated by the environment; population fluctuations tend to be more pronounced in less diverse environments, for example, large areas of homogeneous plant cover, which are mostly replanting plots [7]. In forest stands, which are often similar to natural conditions, high species diversity and potential regulatory feedback (e.g., natural predators) tend to limit population explosions [8, 9].

Mountain virgin forest stands were heavily endangered by air pollution, which is the main cause of dieback particularly in forests of upper-most locations in mountains of Central Europe [8]. Large clearings occurred also in Beskydy Mountains. One of the best preserved areas, where emission clearing occurred in relatively minor extent, was Kněhyně Natural Nature Reserve. But even there, on emission clearing, field vole (*Microtus agrestis*) was dominant rodent species causing damage to forest regeneration [8].

Two species of voles are mainly responsible for a variety of forms of damage especially bark damage to forest trees in mountains. The most important are field voles (*Microtus agrestis*) which prefer open grassy habitats and cause most of the damage to newly planted or replanted forest area [3, 10, 11]. In Central Europe, the field vole was not initially identified as a regeneration pest species since air pollution was more strongly implicated in forest damage [12, 13]. Bank voles (*Myodes glareolus*) are more associated with forest stands and are more likely to affect natural regeneration of woodlands or small replanted areas. In winter, this species also feeds on bark and tree buds, sometimes even above the snow level and vole damage can reach the tops of the young trees [11, 14]. Its diet also includes the herb component, but during the period when the tree seed crop is available, seeds form the dominant component and strongly influence population growth [15, 16].

The aims of the paper are (1) to evaluate the negative effects of voles on natural forest regeneration as well as on replanting and (2) to evaluate factors which may influence rodent damage. We presume that the vole damage would be related to their densities which are influenced by environmental conditions. Other important factor was the vole diet preferences of the tree bark and tree age. In mountains, snow conditions should be also taken in account.

## 2. Material and Methods

### 2.1. Study Area

Research was concentrated in a supra-regional protected area biocentre of 1150 ha in the upper area of the Moravian-Silesian Beskydy Mountains. Within this the core area comprises the Kněhyně National Nature Reserve (NNR) at an altitude of 940 to 1257 m, composed of 195 ha of natural and seminatural beech (*Fagus sylvatica*) and Norway spruce (*Picea abies*) forest stands. Spruce-rowan stands prevail in the summit areas, comprising 23% of the area. Among regeneration stage stands, rowan of all age classes was present. At lower altitudes at protective or buffer zone of NNR, secondary beech stands and spruce were predominant with some planted trees on clearings [17].

#### 2.1.1. Description of Localities

Monitoring plots were laid out on characteristic areas of Kněhyně NNR, the remnant of natural forest stands with the rich spectrum of forest types with typical fauna and flora, and at its protective, buffer area. Protective area of forest is isolating NNR from direct influence of commercial forestry and its management is more close to nature forestry.

Within the plots (homogenous parts of forest described below), small rodent species were collected and the damage to trees was monitored.

SM1: core area of NNR. 60-year-old closed canopy rowan forest with a spruce admixture (altitude 1140 m).
SM2: emission clearing caused by air pollution at the mountain top in NNR (about 1 ha) with natural regeneration of beech and rowan (altitude 1220 m).
SM3: core area of NNR. SE slope of the hill, beech-spruce forest with gaps in canopy (altitude 1120 m).
SM4: protective area, closed canopy 200 years beech forest with admixture of fir (altitude 940 m).
SM5: core area of NNR, closed canopy spruce forest (up to 300 years old) (altitude 1200 m).
SM6: protective area, open canopy forest artificial beech planting 5 years of age with admixture of broad leaved and coniferous trees (altitude 1000 m).
SM7: protective area, open canopy forest artificial beech planting 7 years of age with admixture of broad leaved and coniferous trees (altitude 940 m).

### 2.2. The Structure and Dynamics of Vole Species

Small terrestrial mammals were caught in snap traps baited with fried wicks (soaked in fat and flour), exposed for three nights and checked every morning. Traps were set at 3-meter intervals in a line of 100 traps on 5 monitoring plots. Traps were installed in two additional plots of protective area one year later (SMs 6 and 7). Trapping was carried out three times a year (May, July, October) from 1998 to 2000, that is, a total of 13774 trap nights (i.e., how many traps were lead for how many days = nights). *I* index of abundance (*I*) was calculated according to the formula *I* = 100 × *n*/*P*, where *n* is the number of animals captured in a particular trapping session and *P* is the number of trap nights.

### 2.3. Damage to Trees

Together with the rodent's collection, young trees were checked to rodent impact on bark. The first observation of damage was in spring 2000 and within the localities of small mammal species trappings, young trees were controlled for rodent damage in homogenous and similar type of forest. As a bank vole population peaked in autumn 2000, another checking was done in spring 2001 with minimum of damaged trees (see Table 1). Young individuals of six tree species were checked for bark damage (beech: *Fagus sylvatica*, rowan: *Sorbus aucuparia*, sycamore maple: *Acer pseudoplatanus*, sallow: *Salix caprea*, Norway spruce: *Picea abies,* and silver fir: *Abies alba*) in research area. The young trees were divided into two basic categories.

Category 1 (C1): young trees from artificial plantations and natural regeneration with a stem diameter of up to 2 cm, which were usually no higher than 1 m. Damage was scored as: 0: no bark removed; 1: small patches of bark removed (up to 1 cm^2^); 2: larger extent of bark removed; 3: girdling (bark removal around the tree stem cause tree mortality). Scores 1 and 2 were in evaluation combined into a single one.

Category 2 (C2): older young trees with a stem diameter over 2 cm (height individually measured). Damage was scored as: 0: no bark removed; 1: branches debarked only; 2: bark removed from up to 1 dm^2^; 3: bark removed from more than 1 dm^2^; 4: complete girdling.

In total, 10 006 young individuals of six tree species were inspected.

### 2.4. Factors Affecting Impact of Rodents

In selected plots, the microhabitat in a diameter about 1 m area around each individual young tree C1 category specimen, controlled for bark damage, was recorded. An estimate was made of the percentage cover by dominant species in the herb layer. The cover of *Rubus* sp. and grasses and herbs as a group was categorized as: 1 (less than 25%), 2 (from 25 to 50%), 3 (50 to 75%), and 4 (75 to 100%). The canopy of the trees above the sampling plots was categorized as closed canopy, broken canopy, and open canopy.

As in autumn of 1999 was the only “seed year” of beechnuts (seeds of *Fagus sylvatica *L.) in our research period, its crop was estimated. At localities within the NNR (with a dominance of old beech), the fresh biomass of beechnuts was estimated in 30 plots at 0.25 m^2^. Viable (not damaged and wormy) beechnuts were selected with a mean weight of 2.5 g ± SD 0.3. About 40 beechnuts/m^2^ (i.e., 400 000 seeds/ha) were found under fertile trees.

We evaluated snow conditions (duration of the snow layer) according to the Central Hydrological and Meteorological Institute, for the period of research (1997 to 2001) in the area of NNR.

### 2.5. Statistical Evaluation

The independence of damage frequency by year (i.e., density of voles) and category of trees was tested using goodness-of-fit (GOF) tests with contingency tables. In case of spruce and fir, these were not computed as expected frequencies were lower than 5.

Differences in frequency of damage between various categories of trees and between various localities were also evaluated by goodness-of-fit (GOF) tests with contingency tables.

Differences in snow layer duration (days) in winters of 1999/2000 and 2000/2001 were evaluated by a Wilcoxon matched pair test.

The influence of tree canopy (close, broken, and open) and cover of *Rubus* sp., grasses, and herbs on the degree of bark damage was tested with a generalized linear model (GLM), that is, “multiway analysis of variance” for ordinal multinomial data (10 006 plots). All statistical evaluations were done in Statistica 6.0 [18].

## 3. Results

### 3.1. The Structure and Dynamics of Vole Species

In 1998–2000, small terrestrial mammals were monitored. Of the species, which may be responsible for damage to trees, bank vole (*Myodes glareolus*) (23.9% of all species) and field vole (*Microtus agrestis*) (11.4%) were the dominant.

**Figure 1:**
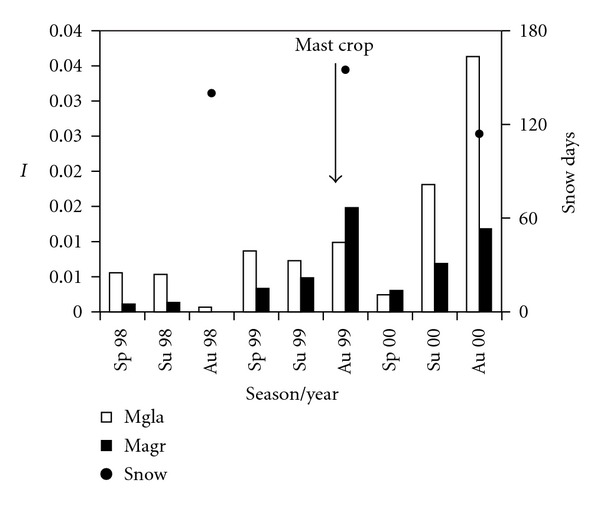
Fluctuation of the relative abundance (*I*) of vole species in the NNR. (Mgla: *M. glareolus;* Magr: *M. agrestis*). ↓ good mast crop; ● number of days with snow cower/year.

The abundance of vole species-fluctuated with minimum numbers on monitored plots in 1998 and a synchronous increase in the autumn of 1999 when field vole reached its maximum. During 2000, bank vole reached its population maximum (Figure 1).

There were species specific preferences of the habitat. Bank vole steadily increased abundance in all localities under study with highest population increase in old beech forest (SM4, peak in autumn 2000). An increase in the relative abundance of field voles was noted particularly in 1999 in open plots with natural and artificial regeneration (emission clearing: SM2, plantings: SM6, SM7).

### 3.2. Damage to Trees

As a great rodent damage occurred in spring 2000, some 5, 227 individuals of six species of trees were controlled. Damage caused during the winter (1999/2000) was found in 16.4% of trees in category C1 and 23.5% in category C2 (20% in average; Table 1). Bark damage in broadleaved trees occurred mostly at the base of the stem at ground level up to a height of 0.5 m (typical field vole damage) but in some shrubby beech trees, branches and tops were damaged up to a height of about 3 m and 20 cm diameter (bank vole damage [14]). The damaged trees were not evenly distributed but in clusters. Tree mortality caused by bark girdling was found only in beech and rowan (2.26% individuals in category C1 and 4.96% individuals in category C2). Among coniferous species (spruce and fir), no stems were damaged, but only bark from branches and growth apexes. The bark of deciduous trees was more attractive to voles (22% damaged) than conifers (8%).

As the abundance of the bank vole was high in the autumn 2000, and we predicted the impact on the forest regeneration by this species, in spring 2001, 4, 779 individuals of the same tree species were checked for bark damage in the same sites as in 2000. Contrary to our expectation, the damage frequency in broadleaved trees was significantly lower, and none of the coniferous species was damaged (Table 1).

### 3.3. Factors Affecting Impact: Tree Size (Age) and Environmental Effects

Since the negligible damage occurred in 2001, only data from winter 2000 were analysed to determine the factors influencing the bark damage caused by rodents. Damage was found both on trees C1 and C2. The frequency of damage in particular category, however, depended upon the tree species (Table 1). In beech, older individuals were more damaged (C1 < C2, GOF test, *P* < 0.001). The highest frequency of damage was noted in trees with stem diameter from 5 to 10 cm and height about 2 m. In rowan, the opposite applied (C1 > C2, GOF test, *P* < 0.001); the highest damage occurred in small individuals (C1) growing in clusters. In sycamore maple, bark damage was noted only in younger individuals (C1) and in sallow and spruce, only in older individuals (C2).

The risk of damage to young trees was markedly dependent on the type of biotope (microhabitat). In the aggregate sample of beech, rowan and sycamore of category C1 from 2000, the effects of microhabitat (herbs, grasses, and *Rubus* cover), and tree canopy were tested with respect to the degree of bark damage. A GLM for ordinal multinomial data showed that bark damage was positively influenced by absence of tree canopy (*χ* ^2^ = 86.94, *P* < 0.001) and a higher cover of grasses (*χ* ^2^ = 44.04, *P* < 0.001). There was lower effect of herb cover (*χ* ^2^ = 11.99, *P* = 0.002) and no significant effect of *Rubus* sp. cover (*χ* ^2^ = 2.55, *P* = 0.110).

**Figure 2:**
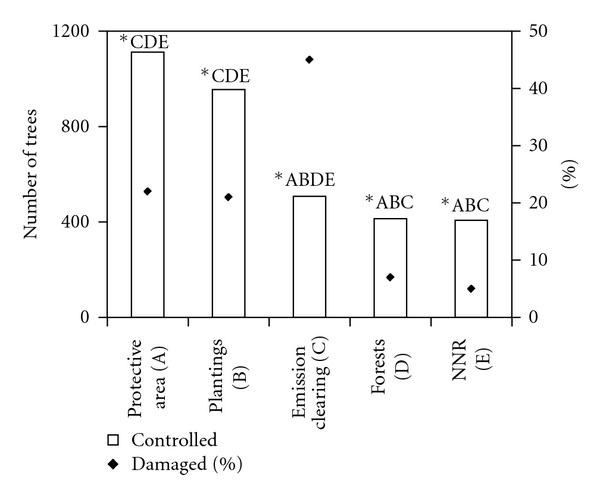
Influence of various forest types (management) on the rodent damage. Different letters above the graphs indicate statistical significant differences between habitats (*P < 0.05).

Damage occurred significantly more in open localities (emission clearing: SM2, plantings: SM6, SM7) with a higher cover of grasses, which also had higher populations of field voles in autumn 1999. Much more damaged were the trees in the emission clearing if compared with other sample plots in the NNR (*χ* ^2^ = 145.37, *P* < 0.001). Natural regeneration in NNR was less damaged if compared to planted trees (*χ* ^2^ = 55.89, *P* < 0.001). A comparison of damage to trees in NNR core area and that of its protective area resulted in lower damage in the core area (*χ* ^2^ = 21.15, *P* < 0.001) (Figure 2).

Vole damage to young trees originates in winter, and influence of snow conditions was evaluated by comparing snow duration (days). If snow condition in winter 1999/2000 (peak abundance of the field vole and damage to trees) was compared to winter 2000/2001 (peak abundance of the bank vole and no damage to trees), significant differences would be found (Wilcoxon *z* = 2.37; *P* = 0.01) (Figure 1).

## 4. Discussion

During the study, abundance of rodent species in NNR fluctuates from minimum to maximum with specific preference of the stand type. Bank vole reached the high abundance in old beech forest a year after good beech mast crop. The highest abundance of the field vole was in open plots with tree regeneration. Control of rodent damage to trees in spring 2000 revealed bark damage on 20% of trees with the peak abundance of the field voles in combination with long winter. Contrary to our expectation, high abundances of the bank vole and shorter winter resulted in low impact on tree regeneration. Tree mortality caused by bark girdling was referred in 7% of the trees. Deciduous trees were more damaged in contrast to low damage to coniferous trees. Also, planted tree species composition and its age influenced the impact intensity. The main factors affecting the degree of impact were the higher population densities of the field voles, which preferred open and grassy plots. Clearings caused by air pollution and replanting plots develop prepositions for higher impact of vole species in comparison to the natural regeneration in close to nature habitats [3, 12, 19]. The other important factor in mountains was snow cover, which is, in open plots higher, more compact and longer lasting.

In National Nature Reserve Kněhyně, clearings were caused by air pollution of relatively minor extent (area about 1 ha). High diversity of community was confirmed [8] in study area. Under close to nature conditions, multiyear fluctuation changes in the abundance of monitored rodents do not occur so as in large area of clearings after air pollution [5]. Even this under special conditions, high damage to young trees was observed.

In according to our results, Gill [11] in a review of damage caused by small mammals to the forest confirmed the influence of many factors on its intensity. Deep studies of factors affecting bark damage by voles to young trees in Europe have been conducted by Hansson (e.g., food [20], snow cover [21], bark chemical content [22, 23], influence of habitat [23–25], review articles [14, 26]). 

Bark damage was, as in our study, mostly related to winter period. Bark eating by folivores is strongly correlated to pronounced population fluctuation in autumn previous to winter [2, 3]. Bark is not the preferred food of voles; bark is nutritionally inferior to the normal diet of voles [27]. It is known that the bark of deciduous species is preferred to that of coniferous trees [28, 29].

Our research concentrated on regeneration of the beech. Most damage was to trees about 2 m high (C2). In Scandinavia, vole damage was reported to be most common in trees about 125 cm tall [11]. Growth-related factors appear important in determining which beech trees suffer the greatest damage. Young beech trees grow side branches very early, and, thus, snow is not so compact under such trees. In NNR naturally regenerated beech trees grow in groups, providing good conditions for wintering voles with enough bark and cover for many months. This is another reason why beech trees of category C2 suffered more bark damage.

Rowan is one of the most important tree species in the mountain spruce forest ecosystem (*Sorbeto-Piceetum*) in central Europe contributing to forest stability and natural regeneration [30]. The susceptibility of rowan to bark injury by voles was found to be strongly size dependent, with the highest rate of damage occurring to the smaller (C1) individuals. Certain chemicals (e.g., P, Na, Ca, K) can influence diet preferences. Hansson [22] found that the degree of bark consumption correlated with its mineral content. The food needs to contain the right proportions of these chemicals in order to be utilized by the animal. Wöhlbier and Lindner [31] found that the content of trace elements is significantly higher in bark than in herbs and grasses. Thus, even though it is not a preferred diet item, bark can help to support vole through the winter when other food is not available [11].

High snow cover (at the top of mountains up to 3 m) enables voles to access higher branches, as under the trees snow is not so compact. In agreement with us, Baxter and Hansson [26] found young trees with trunks up to a diameter of 10 cm were often damaged. Field voles can reach the tree trunk as high as the depth of snow allows and remove the bark. Bank voles climb up to the tree crown for food and can therefore damage bark high above the snowline [14]. Stable snow cover is very beneficial to voles, as it insulates them from extreme cold temperatures and provides excellent protection against many predators. What is detrimental is when temperatures repeatedly climb above zero during winters, especially if this is accompanied by rainfall. This will cause melting water to seep to ground level, and when temperatures again decrease, this water will free up and both encase ground vegetation with ice, inhibit voles from moving in the snow pack [32]. This happen usually when snow cover duration is long lasting. This was confirmed by Hansson and Henttonen, [21] who found snowy winters together with high population densities caused voles to have a negative impact on forest regeneration. The amount of snow and its configuration might vary between years due to changing weather patterns as in our mountain locality.

In our study, grassy clearings provide a suitable habitat for a field vole. By T. P. Sullivan and D. S. Sullivan [19], vole numbers were higher on sites sown with pasture grasses and herbs. There was a significant positive relationship of tree mortality and abundance of voles (*Microtus*) across a relatively wide geographic area. This was also confirmed by Birney et al. [33]. The destruction of vegetation with herbicides, grazing or cutting is widely recognized control techniques for many rodent species [11]. Grass control in tree microhabitats is known to decrease vole damage [34]. Herb layer control such as vegetation removal in reforestation plots is costly and in mountain terrain difficult to manage. In core area of Kněhyně NNR, there is minimal or no forest management, so natural development and regeneration is important.

Impact of the tree debarking depends on the wound extension. Girdling causes tree mortality. In our case, it was 7.22% of the all controlled trees. The majority of trees appear to survive partial debarking and exceptionally even if 90% of the circumference is debarked. Small stems are more easily girdled then larger and death of younger trees is more common [11]. The impact of debarking of various extensions may cause many other grow complications as tree deformation, rot developing, breakage of tree in wound, and so on.

## 5. Conclusion

In mountain forests, a negative impact of voles on regeneration arose in the presence of specific factors related primarily to higher population densities (particularly for field vole) in open habitats (emission clearings and artificial plantings) in combination with longer duration of snow cover in winter. As significantly lower damage was found on natural regenerated young trees in natural conditions, it should be supported in silviculture management. In addition, voles prefer planted trees, with their nursery fertilization regime and enhanced palatability and nutrition, to wildlings arising from natural regeneration [35]. Young trees on open and grassy plots may be under higher vole-damage impact. Grass and weed control in tree microhabitat may prevent or reduce rodent impact.

## Figures and Tables

**Table 1:** Controlled trees (C1: younger; C2: older) in two successive years (after winter 2000 and 2001) and their rodent damage (%).

Category	Tree	Spring 2000			Spring 2001			GOF test
		Controlled	Damaged	%	Controlled	Damaged	%	
C1	Beech	1485	217	14.61	1461	7	0.48	<0.001
	Mountain ash	906	248	27.37	907	4	0.44	<0.001
	Maple	218	44	20.18	231	1	0.43	<0.001
	Spruce	403	0	0.00	373	0	0.00	(a)
	Fir	104	1	0.96	100	0	0.00	(a)

C2	Beech	1046	392	37.48	829	1	0.12	<0.001
	Mountain ash	345	33	9.57	389	2	0.51	<0.001
	Willow	600	50	8.33	362	0	0.00	<0.001
	Spruce	120	20	16.67	127	0	0.00	<0.001

(a) Not computed (expected frequency lower than 5).
